# Outcome of Transcatheter Aortic Valve Replacement for Pure Native Aortic Regurgitation in Patients with Pulmonary Hypertension

**DOI:** 10.31083/j.rcm2508307

**Published:** 2024-08-23

**Authors:** Da-wei Lin, Zi-long Weng, Jia-ning Fan, Yu-liang Long, Li-hua Guan, Wen-zhi Pan, Da-xin Zhou, Jun-bo Ge

**Affiliations:** ^1^Department of Cardiology, Zhongshan Hospital, Fudan University, 200032 Shanghai, China; ^2^National Clinical Research Center for Interventional Medicine, 200032 Shanghai, China

**Keywords:** pure native aortic regurgitation, transcatheter aortic valve replacement, pulmonary artery hypertension, retrospective

## Abstract

**Background:**

In recent years, transcatheter aortic valve replacement (TAVR) has emerged as a pivotal treatment for pure native aortic regurgitation (PNAR). Given patients with severe aortic regurgitation (AR) are prone to suffer from pulmonary hypertension (PH), understanding TAVR’s efficacy in this context is crucial. This study aims to explore the short-term prognosis of TAVR in PNAR patients with concurrent PH.

**Methods:**

Patients with PNAR undergoing TAVR at Zhongshan Hospital, Affiliated with Fudan University, were enrolled between June 2018 to June 2023. They were categorized based on pulmonary artery systolic pressure (PASP) into groups with or without PH. The baseline characteristics, imaging records, and follow-up data were collected.

**Results:**

Among the 103 patients recruited, 48 were afflicted with PH. In comparison to PNAR patients without PH, the PH group exhibited higher rates of renal dysfunction (10.4% vs. 0.0%, *p* = 0.014), increased Society of Thoracic Surgeons scores (6.4 ± 1.9 vs. 4.7 ± 1.6, *p* < 0.001), and elevated Nterminal fragment of pro–brain natriuretic peptide (NT-proBNP). Transthoracic ultrasound examination revealed that patients with PH displayed lower left ventricular ejection fraction, larger left ventricle dimension, and more frequent moderate to severe tcuspid regurgitation (TR). Following TAVR, both groups experienced significant reductions in PASP, mitral regurgitation (MR) and TR. There were no significant differences in the incidence of postoperative adverse events in patients with or without PH.

**Conclusions:**

We found TAVR to be a safe and effective treatment for patients with PNAR and PH, reducing the degree of aortic regurgitation and PH without increasing the risk of postoperative adverse events.

## 1. Introduction

Pure native aortic regurgitation (PNAR) arises from intrinsic valve leaflet abnormalities, aortic root dilation, or geometric distortion, or a combination thereof, resulting in retrograde blood flow into the left ventricle during diastole. The prevalence of aortic regurgitation (AR) is positively correlated with age, as individuals over the age of 70 have a diagnosis rate exceeding 2% and face a poorer prognosis [1, 2, 3, 4]. While surgical aortic valve replacement (SAVR) is the preferred treatment for AR, the one-year mortality rate varies from 10% to 20% [5]. Additionally, SAVR may not be an option for patients of advanced age or those with compromised heart function.

Transcatheter aortic valve replacement (TAVR) is an important treatment option for severe aortic valve stenosis (AS) in individuals at high surgical risk, offering advantages including faster recovery and reduced trauma [5, 6]. Advances in TAVR have broadened its application to include lower-risk patients, thereby expanding the eligible patient base. However, its application to PNAR treatment remains limited, as the absence of valvular calcium presents significant challenges, including increased risks of valve embolization, migration, and para-valvular leak.

Nearly 20% of individuals with PNAR also experience pulmonary hypertension (PH), with 10%–16% facing severe conditions [7, 8], a prevalence surpassing that observed in individuals experiencing severe stenosis or aortic valve disease. This disparity arises from blood regurgitation into the left ventricle during diastole in cases of AR, increasing the pressure load to the left ventricle, eventually resulting in PH. While TAVR has proven effective for AS patients with PH [9, 10], its performance in PNAR patients with concomitant PH remains unknown. This gap in knowledge prompted our study, aiming to evaluate the safety and efficacy of TAVR for PNAR patients with PH.

## 2. Subjects and Methods

### 2.1 Research Subjects

For this study, we recruited patients diagnosed with PNAR and implanted with VenusA-Valve (Venus Medtech, Hangzhou, China) or VitaFlow valve (Microport, Shanghai, China) at Zhongshan Hospital, Fudan University, from June 2018 to June 2023. All patients were considered unsuitable for surgical valve replacement after a comprehensive evaluation. In addition, all patients had a follow-up duration of ≥6-month and underwent cardiac ultrasound examinations. Exclusion criteria were as follows: (1) patients who had failed bioprosthetic valve surgery; (2) peak aortic valve pressure gradient measured by pre-TAVR echocardiography greater than 20 mmHg; (3) concomitant hypertrophic obstructive cardiomyopathy; (4) concomitant left ventricular thrombus or infective endocarditis. This study was approved by the Ethics Committee of Zhongshan Hospital, Fudan University (number: B2020-039), and all patients were informed and signed a consent form.

### 2.2 Patient Classification and Data Collection

The PH classification was as follows. Based on the pulmonary artery pressure grading criteria from previous studies [11], patients were divided by the presence of PH (pulmonary artery systolic pressure, PASP ≥35 mmHg) or the absence of PH (PASP <35 mmHg) according to the PASP measured by echocardiography. Clinical information of patients was collected from medical records and the catheterization laboratory information system for retrospective analysis. We documented the baseline patient information taken prior to surgery, such as hypertension, diabetes, pulmonary arterial hypertension, atrial fibrillation, heart failure, and renal insufficiency. Patients underwent multi-detector computed tomography (CT) and echocardiography examinations, and postoperative follow-up echocardiography. The parameters such as left ventricular ejection fraction, left atrial and left ventricular dimensions, degree of aortic valve regurgitation, and mean transvalvular pressure of the aortic valve were recorded. Grading of regurgitation severity was based on the valve regurgitant jet area obtained from echocardiography: Grade 0: No regurgitation or trivial regurgitation; Grade 1: Mild regurgitation; Grade 2: Moderate regurgitation; Grade 3: Severe regurgitation.

### 2.3 Treatment and Follow-up

All patients underwent TAVR via intravenous anesthesia by the structural heart disease surgery team in the Department of Cardiology, Zhongshan Hospital. Patients had a post operative follow-up visit at the outpatient department after 30 days and 6 months, with transthoracic echocardiography (TTE) and electrocardiography (ECG) examinations performed, and perioperative endpoint events recorded. The definition of clinical outcomes followed the Valve Academic Research Consortium-3 (VARC-3) criteria [12]. The primary endpoints included all-cause death and cardiovascular death. The secondary endpoints included TAVR-related complications, such as myocardial infarction, major bleeding events, major vascular complications, acute kidney injury, stroke, endocarditis, new-onset atrial fibrillation, implantation of a new pacemaker, coronary artery obstruction, mild paravalvular leak, and rehospitalization.

### 2.4 Statistical Analysis

Statistical analysis of the data was conducted using STATA (version 15.1; Stata Corporation, College Station, TX, USA). Continuous variables were expressed as mean ± standard deviation (SD) and were analyzed using either the Student’s *t*-test or the Mann-Whitney U test. Continuous variables in the PH and non-PH groups were compared using the Student’s *t*-test or the Mann-Whitney U test while categorical variables were compared using the chi-square or Fisher’s exact test. To compare baseline and post-procedure follow-up periods, continuous variables were analyzed using the paired Student’s *t*-test, whereas categorical variables were compared using the Wilcoxon signed-rank test. All statistical tests were two-sided, and significance was defined as *p* values < 0.05.

## 3. Results

### 3.1 Comparison of Baseline Clinical Information and Imaging Data of Patients in the Groups of Patients with or without PH

In this study, 103 patients were enrolled, including 62 males and 41 females with a mean age of (72.1 ± 8.1) years. Notably, 48 (46.6%) of these patients suffered from PH. Clinical data revealed significant differences between patients with PNAR with and without PH. Patients with PH were more likely to be classified in New York Heart Association (NYHA) functional classes III and IV (93.8% vs. 76.4%, *p* = 0.015) and were more frequently diagnosed with renal dysfunction (10.4% vs. 0%, *p* = 0.014) with higher level of serum creatinine (122.2 ± 113.2 vs. 82.4 ± 22.4, *p* = 0.012). Additionally, they exhibited a higher Society of Thoracic Surgeons (STS) score (6.4 ± 1.9 vs. 4.7 ± 1.6, *p* < 0.001) and elevated serum levels of Nterminal fragment of pro–brain natriuretic peptide (NT-proBNP) (2542.1 ± 2290.2 vs. 1050.4 ± 1611.7, *p* < 0.001). However, no significant differences were observed in baseline body mass index (BMI), hypertension, diabetes, atrial fibrillation, chronic lung disease, chronic kidney disease, previous percutaneous coronary intervention, previous pacemaker implantation, or creatinine between the two groups (Table 1).

**Table 1.** **Baseline demographic and clinical characteristics of PNAR patients with and without PH**.

Patient characteristics		Without PH Group (n = 55)	PH Group (n = 48)	*p* value
Age, yrs		72.9 ± 7.0	71.2 ± 9.2	0.28
Male		31 (56.4%)	31 (64.6%)	0.40
Body mass index (kg/m^2^)		23.1 ± 2.2	22.6 ± 2.9	0.33
Hyperlipidemia, %		21 (38.2%)	14 (29.2%)	0.34
Hypertension, %		40 (72.7%)	33 (68.8%)	0.66
Diabetes mellitus, %		3 (5.5%)	4 (8.3%)	0.56
Atrial fibrillation, %		11 (20.0%)	13 (27.1%)	0.40
Coronary artery disease, %		10 (18.2%)	10 (20.8%)	0.73
Previous PCI, %		8 (14.5%)	6 (12.5%)	0.79
COPD, %		8 (14.5%)	5 (10.4%)	0.53
PPM, %		2 (3.6%)	4 (8.3%)	0.31
Peripheral vascular disease, %		0 (0%)	1 (2.1%)	0.28
Renal insufficiency, %		0 (0%)	5 (10.4%)	0.014
Symptom, %		21 (38.2%)	26 (54.2%)	0.10
	Left bundle branch block, %	4 (7.3%)	2 (4.2%)	0.50
	Right bundle branch block, %	3 (5.5%)	2 (4.2%)	0.76
Atrioventricular block, %		12 (21.8%)	5 (10.4%)	0.12
NYHA functional class III or IV, %		42 (76.4%)	45 (93.8%)	0.015
Serum creatinine (mg/dL)		82.4 ± 22.4	122.2 ± 113.2	0.012
NT-proBNP (ng/pL)		1050.4 ± 1611.7	2542.1 ± 2290.2	<0.001
STS risk score		4.7 ± 1.6	6.4 ± 1.9	<0.001

Abbreviations: PNAR, pure native aortic regurgitation; PH, pulmonary hypertension; COPD, chronic obstructive pulmonary disease; PCI, percutaneous coronary intervention; PPM, permanent pacemaker; NYHA, New York Heart Association; STS, Society of Thoracic Surgeon; NT-proBNP, N-terminal fragment of pro–brain natriuretic peptide.

TTE examination further differentiated the two groups. Patients with PH group exhibited significantly higher PASP (44.3 ± 7.6 vs. 30.7 ± 2.6, *p* < 0.001), lower left ventricular ejection fraction (LVEF) (51.9 ± 12.1 vs. 56.7 ± 10.4, *p* = 0.030), larger left ventricular end-diastolic dimension (LVEDd) (61.3 ± 10.2 vs. 55.2 ± 4.7, *p* < 0.001), left ventricular end-systolic dimension (LVEDs) (45.7 ± 10.5 vs. 38.4 ± 6.3, *p* < 0.001), along with a thicker end-diastolic left ventricular posterior wall (10.8 ± 1.5 vs. 10.0 ± 1.3, *p* = 0.005), while no notable difference was found in inter-ventricular septum thickness. Furthermore, a higher proportion of patients in the PH group exhibited moderate to severe tricuspid regurgitation (TR) (37.5% vs. 20.0%, *p* = 0.049). However, no significant differences were noted between these two groups in terms of mitral regurgitation (MR), mean transvalvular pressure gradient, effective regurgitant orifice area, annulus area, annulus perimeter, or the largest diameter of the ascending aorta (Table 2).

**Table 2.** **Baseline echocardiographic and imaging parameters in PNAR patients with and without PH**.

		Without PH Group (n = 55)	PH Group (n = 48)	*p* value
Echocardiography				
	LVEF (%)	56.7 ± 10.4	51.9 ± 12.1	0.030
	LVEDd (mm)	55.2 ± 4.7	61.3 ± 10.2	<0.001
	LVEDs (mm)	38.4 ± 6.3	45.7 ± 10.5	<0.001
	LVPWd (mm)	10.0 ± 1.3	10.8 ± 1.5	0.005
IVS (mm)		10.9 ± 1.6	11.0 ± 1.4	0.71
Severe aortic regurgitation, %		55 (100%)	48 (100.0%)	-
Aortic valve peak velocity (m/s)		1.9 ± 0.7	2.0 ± 0.8	0.69
Mean valve gradient (mmHg)		9.0 ± 7.7	9.0 ± 6.3	0.99
Effective orifice area (cm^2^)		2.8 ± 0.7	2.7 ± 1.0	0.59
Moderate to severe MR		13 (23.6%)	16 (33.3%)	0.28
Moderate to severe TR		11 (20.0%)	18 (37.5%)	0.049
PASP, mmHg		30.7 ± 2.6	44.3 ± 7.6	<0.001
Computed tomography				
	Aortic annulus area (mm^2^)	505.7 ± 75.5	533.1 ± 72.5	0.064
	Aortic annulus perimeter (mm)	83.3 ± 6.3	84.2 ± 7.1	0.46
	Largest diameter of ascending aorta (mm)	34.6 ± 4.3	36.3 ± 4.4	0.050

Abbreviations: LVEF, left ventricular ejection fraction; LVEDd, left ventricle end-dimension diastole; LVEDs, left ventricle end-dimension systole; LVPWd, left ventricular posterior wall thickness at end-diastole; IVS, interventricular septal thickness; MR, mitral regurgitation; TR, tricuspid regurgitation; PNAR, pure native aortic regurgitation; PH, pulmonary hypertension; PASP, pulmonary artery systolic pressure.

### 3.2 Comparative Analysis of Cardiac Function and Echocardiographic Outcomes Pre- and Post-TAVR in Patients with or without PH

**Fig. 1.**
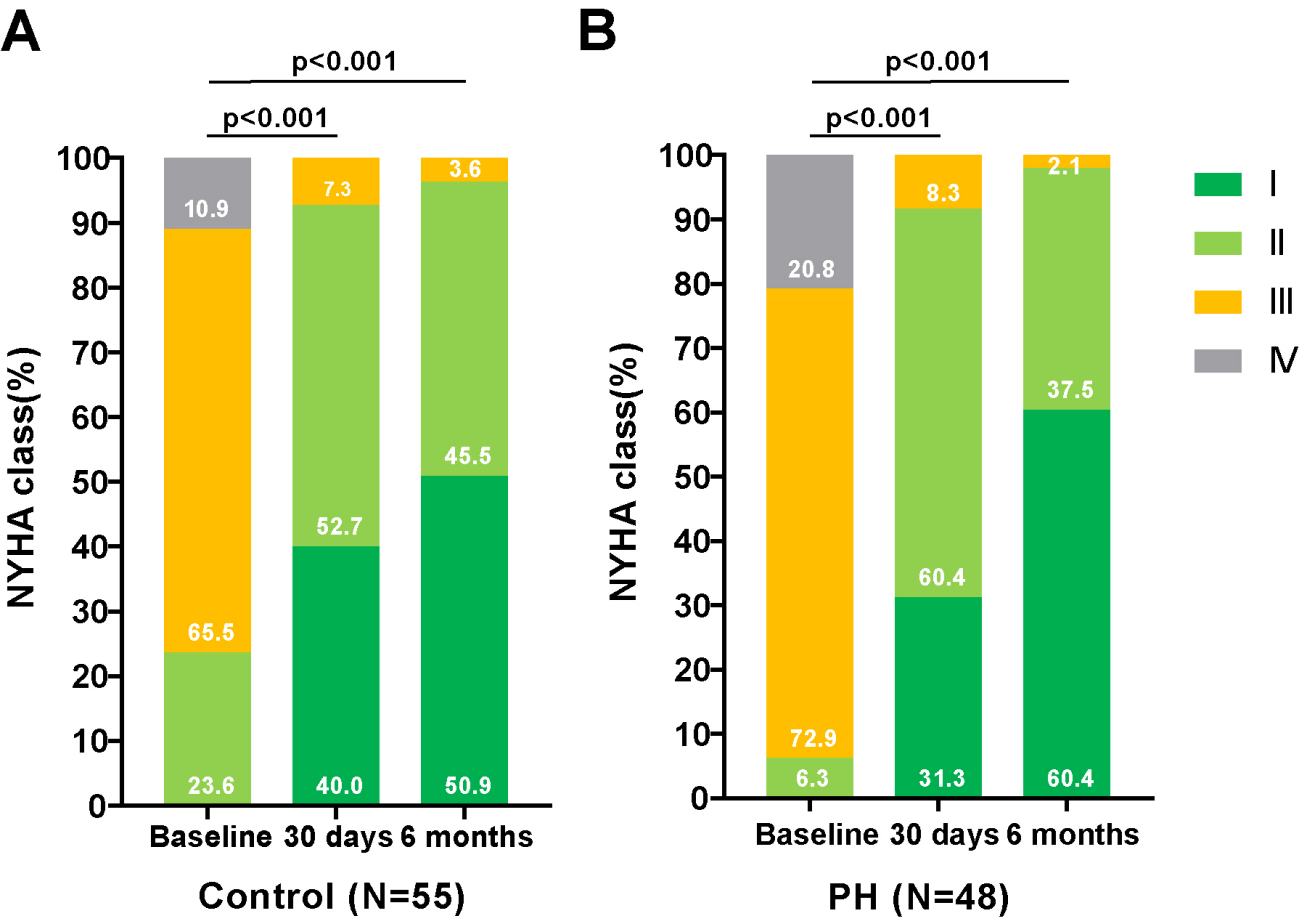
**Changes in the NYHA functional classification in PNAR patients Pre- and Post-TAVR, with and without PH**. Assessment of the NYHA functional class changes in patients with PNAR undergoing TAVR, categorized by the presence or absence of PH. (A) illustrates the progression in patients without PH, while (B) details those with PH. These comparisons highlight the procedural impact on cardiac functional status over the follow-up period. Abbreviations: PH, pulmonary artery hypertension; TAVR, transcatheter aortic valve replacement; NYHA, New York Heart Association; PNAR, pure native aortic regurgitation.

**Fig. 2.**
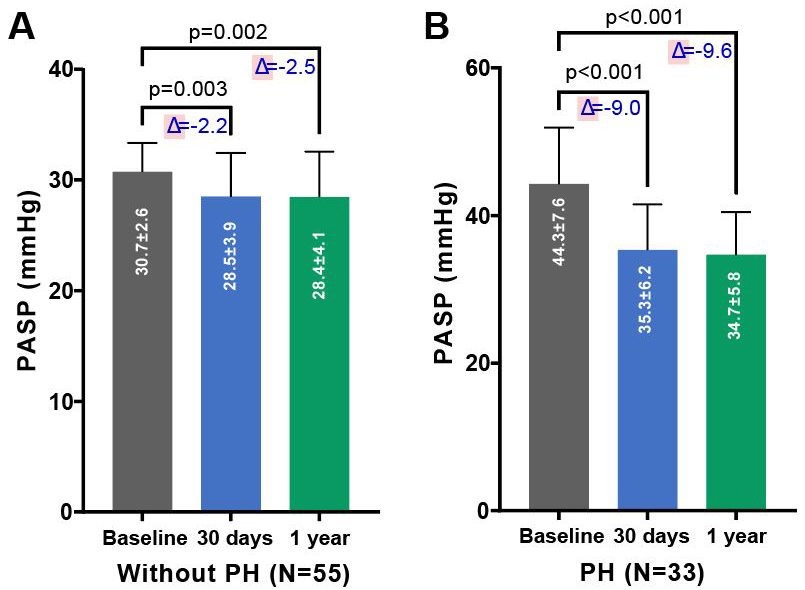
**Changes in PASP pre- and post-TAVR in PNAR patients, segregated by PH status**. Comparison of PASP before and after TAVR in patients with PNAR, differentiated by the absence (A) or presence (B) of PH. The figure underscores the procedural impact on PASP across both patient categories over the follow-up period, demonstrating the effectiveness of TAVR in modifying hemodynamic parameters. Abbreviations: PH, pulmonary artery hypertension; TAVR, transcatheter aortic valve replacement; PASP, systolic pulmonary artery pressure; PNAR, pure native aortic regurgitation.

As depicted in Figs. 1,2, there was a significant improvement in cardiac function observed in both groups—those with and without PH—at 30 days and 6 months post-TAVR, compared to baseline measurements. This improvement was accompanied by a notable reduction in PASP. As shown in Table 3, compared to baseline, patients with PH demonstrated a significant decrease in PASP (35.3 ± 6.3 vs. 44.3 ± 7.6, *p* < 0.001), average grade of MR (0.9 ± 1.1 vs. 1.7 ± 0.9, *p* < 0.001), and average grade of TR (1.0 ± 1.1 vs. 2.0 ± 1.0, *p* < 0.001) at 30 days post-intervention. These changes persisted significantly at 6 months, with marked decreases in PASP (34.7 ± 5.8 vs. 44.3 ± 7.6, *p* < 0.001), average grade of MR (0.8 ± 1.0 vs. 1.7 ± 0.9, *p* < 0.001), and average grade of TR (0.9 ± 1.0 vs. 2.0 ± 1.0, *p* < 0.001). Furthermore, there was a significantly lower proportion of patients with moderate to severe TR at 6 months compared to baseline, though this difference was not observed at 30 days. For the group without PH, similar significant reductions in PASP, MR, and TR were recorded at both the 30-day and 6-month follow-ups compared to baseline. Additionally, both groups experienced a notable reduction in the proportion of patients with moderate to severe AR during the 30-day and 6-month of follow-ups, while there were no significant changes in LVEF.

**Table 3.** **Comparison of pre- and post TAVR chocardiography parameters in patients with or without PH**.

Echocardiography	Without PH Group (n = 55)				
	Baseline	30 days	*p_1_* value	6 months	*p_2_* value
LVEF (%)	56.7 ± 10.4	55.2 ± 7.8	0.42	56.4 ± 9.8	0.94
PASP, mmHg	30.7 ± 2.6	28.5 ± 3.9	<0.001	28.4 ± 4.1	0.002
Moderate to severe MR, %	13 (23.6%)	3 (5.5%)	<0.001	3 (5.5%)	<0.001
Average grade of MR	1.7 ± 0.9	0.9 ± 1.0	<0.001	0.8 ± 0.8	<0.001
Moderate to severe TR, %	11 (20.0%)	6 (10.9%)	0.11	2 (3.6%)	0.014
Average grade of TR	1.4 ± 0.8	0.9 ± 0.8	<0.001	0.9 ± 0.7	<0.001
Moderate to severe AR, %	55 (100.0%)	2 (3.6%)	<0.001	1 (1.8%)	<0.001
Echocardiography	PH Group (n = 48)				
	Baseline	30 days	*p_1_* value	6 months	*p_2_* value
LVEF (%)	51.9 ± 12.1	50.8 ± 10.7	0.65	52.5 ± 10.9	0.78
PASP, mmHg	44.3 ± 7.6	35.3 ± 6.2	<0.001	34.7 ± 5.8	<0.001
Moderate to severe MR, %	16 (33.3%)	6 (12.5%)	0.015	4 (8.3%)	0.003
Average grade of MR	1.9 ± 1.3	0.9 ± 1.1	<0.001	0.8 ± 1.0	<0.001
Moderate to severe TR, %	18 (37.5%)	7 (14.6%)	0.011	4 (8.3%)	0.003
Average grade of TR	2.02 ± 1.0	1.0 ± 1.1	<0.001	0.9 ± 1.0	<0.001
Moderate to severe AR, %	48 (100.0%)	1 (2.1%)	<0.001	1 (2.1%)	<0.001

*p_1_*: the significance of characteristics in 30 days comparing to those in baseline. *p_2_*: the significance of characteristics in 6 months comparing to those in baseline. Abbreviations: PASP, pulmonary artery systolic pressure; LVEF, left ventricular ejection fraction; MR, mitral regurgitation; TR, tricuspid regurgitation; AR, aortic regurgitation; TAVR, transcatheter aortic valve replacement; PH, pulmonary hypertension.

### 3.3 Analysis of Postoperative in Hospitalization and 6 Months Endpoint Events

There was no significant difference in primary and secondary endpoints in PNAR patients with or without PH who underwent TAVR. Notably, there were no instances of all-cause or cardiac death in either group. The incidence of major in-hospital adverse events was as follows: major bleeding, major vascular complications, stroke, and new-onset atrial fibrillation, new-onset left bundle branch block, new-onset atrioventricular block, permanent pacemaker implants, and mild paravalvular leaks were 2.1%, 0%, 2.1%, 2.1%, 12.5%, 27.1%, 29.2%, and 14.6% in the group with PH and 0%, 0%, 0%, 7.3%, 21.8%, 20.0%, 16.4%, and 5.5% in the group without PH.

During the 6 months follow-up period, hospital admissions were slightly higher in the PH group (3 patients) compared to the control group (1 patient). Additionally, the group with PH exhibited a prevalence of 2.1% for major bleeding events, 4.2% for strokes, 6.3% for permanent pacemaker implants and new-onset atrioventricular block, and 12.5% for mild paravalvular leaks. The group without PH demonstrated a prevalence of 1.8% for new-onset left bundle branch block, 10.9% for new-onset atrioventricular block, 3.6% for new permanent pacemaker implants and 9.1% for mild para-valvular leaks. This data is summarized in Table 4.

**Table 4.** **Comparison of clinical end-points at various follow-up intervals for patients with and without PH Post-TAVR**.

Clinical end-points		In hospital			6 months		
		Without PH group (n = 55)	PH group (n = 48)	*p*	Without PH group (n = 55)	PH group (n = 48)	*p*
Primary endpoints							
	All-cause mortality, %	0 (0%)	0 (0%)	-	0 (0%)	0 (0%)	-
	Cardiovascular mortality, %	0 (0%)	0 (0%)	-	0 (0%)	0 (0%)	-
Secondary endpoints							
	Bleeding event, %	0 (0%)	1 (2.1%)	0.29	1 (1.8%)	1 (2.1%)	0.92
	Major Vascular complication, %	0 (0%)	0 (0%)	-	0 (0%)	0 (0%)	-
	Acute renal failure, %	0 (0%)	0 (0%)	-	0 (0%)	0 (0%)	-
	Stroke, %	0 (0%)	1 (2.1%)	0.29	1 (1.8%)	2 (4.2%)	0.48
	Myocardial infarction, %	0 (0%)	0 (0%)	-	0 (0%)	0 (0%)	-
	New AF, %	4 (7.3%)	1 (2.1%)	0.21	0 (0%)	0 (0%)	-
	New LBBB, %	12 (21.8%)	6 (12.5%)	0.20	1 (1.8%)	0 (0%)	0.35
	New AVB, %	11 (20.0%)	13 (27.1%)	0.42	6 (10.9%)	3 (6.3%)	0.40
	New PPM, %	9 (16.4%)	14 (29.2%)	0.13	2 (3.6%)	3 (6.3%)	0.54
	Endocarditis, %	0 (0%)	0 (0%)	-	0 (0%)	0 (0%)	-
	Mild PVL, %	3 (5.5%)	7 (14.6%)	0.12	5 (9.1%)	6 (12.5%)	0.58
	Re-hospitalization, %	0 (0%)	0 (0%)	-	1 (1.8%)	3 (6.3%)	0.25

Abbreviations: AF, atrial fibrillation; LBBB, left bundle branch block; AVB, atrioventricular block; PPM, permanent pacemaker; PVL, perivalvular leakage; TAVR, transcatheter aortic valve replacement; PH, pulmonary hypertension.

## 4. Discussion

To the best of our knowledge, this study is the first to report the outcomes of TAVR in patients diagnosed with PNAR and PH. The findings indicate that TAVR is a safe option for this patient group, as evidenced by the absence of any significant disparities in all-cause mortality and cardiac mortality between the two groups during hospitalization and throughout a 6-month follow-up period. Moreover, TAVR proves to be an effective intervention for these patients, as it leads to a substantial reduction in AR severity and PASP, while significantly improving cardiac function.

Our study demonstrated a notable decrease in the incidence of AR and PASP in both groups following TAVR. In 6 months post-TAVR, a significant alleviation of AR was observed in 97.9% of the PNAR patients with PH, and in 98.2% of those without PH, indicating the effectiveness of TAVR in both cohorts. Additionally, patients diagnosed with PNAR exhibited a tendency towards higher PASP levels. Previous research has documented that approximately 20% of patients experience PH, with 10–16% classified as severe [7, 8]. This phenomenon is primarily due to increased left ventricular afterload induced by AR, which produces backward spread to left atrium and pulmonary veins. Initially, pulmonary vascular resistance is at normal stage, however chronically elevated left atrial pressure can lead to pulmonary vascular remodeling—a combination of precapillary and postcapillary PH [13, 14].

Furthermore, we found that the increasing PASP in PNAR can be down-regulated after TAVR, meaning that pulmonary vascular remodeling is reversible to some degree. We found a significant decline in the value of PASP in both groups after TAVR, a reduction that was maintained through the 6-month follow-up period. Notably, there was no significant difference in PASP levels between the 6-month and 30-day follow-up. This supports the hypothesis that elevated PASP is a contributing factor to AR severity. A related study by Naidoo DP, *et al*. [14] involving 13 patients undergoing SAVR found that pulmonary vascular resistance decreased from 4.7 ± 3.5 to 1.5 ± 0.8 Wood units, with 10 patients achieving normalized pulmonary artery pressure following the procedure. This parallels our findings, reinforcing the concept that PH is largely reversible following aortic valve replacement. Taken together, observations affirm TAVR’s efficacy in treating patients with PNAR and concurrent PH.

Our study demonstrated that patients with PNAR and PH exhibit compromised cardiac function, characterized by reduced ejection fraction and larger ventricular volume compared to patients without PH. In patients with aortic and/or mitral valve disease, the diagnosis of PH suggests the exhaustion of the compensatory mechanism in the left heart. Additionally, our study noted a higher occurrence of moderate to severe TR among PNAR patients with PH, highlighting the detrimental effect of PH on right heart dynamics.

Encouragingly, following TAVR, the incidence of TR and MR decreased during the initial and 1-month follow-up periods. This outcome indicates that the cardiac remodeling caused by AR and PH can be reversed. Consequently, TAVR appears to be a viable option for patients with AR and also offers potential amelioration for associated MR/TR, providing a comprehensive treatment approach for patients suffering from these complex cardiac conditions.

Our findings affirm the safety of TAVR for patients with PNAR and PH. There were no instances of all-cause death or cardiac mortality in either patient group during hospitalization or within 6-months following the operation. Additionally, through the follow-up, there were no significant differences between groups in the incidence of stroke, permanent pacemaker implantation, mild perivalvular leakage, bleeding events, vascular complications, and new atrial fibrillation events. This contrasts with a large retrospective study in the United States of 915 patients with severe AR after TAVR, which reported a hospital mortality rate of 2.7% and a 1-month post-operative mortality rate of 3.3% [15]. The valve-related complications in this study were 18–19%, with perivalvular leakage (4–7%) and complete atrioventricular block and/or require permanent pacemaker implantation (about 11%) being most common [15]. While the long-term prognosis of patients with severe AR and PH undergoing TAVR has yet to be reported, a study conduct on SAVR in AR patients with PH suggests a higher 10-year survival rate than that in patients without valve replacement (*p* < 0.001) [8]. Given these insights, TAVR emerges as a viable clinical option for individuals with PNAR and PH, extending the potential for enhanced patient outcomes.

### Limitations

Our study is subject to certain limitations that merit consideration. Firstly, it is a retrospective analysis conducted at a single-center, it features a relatively small cohort, and the follow-up period was limited to 6 months. Such constraints may affect the generalizability of the results and underscore the need for larger, multi-center, prospective studies with extended follow-up durations to validate our findings. Secondly, the assessment of PASP was performed using echocardiography, as opposed to right heart catheterization, which is regarded as the gold standard for this measurement. Consequently, the estimated PASP values may lack the precision achievable through catheterization, potentially affecting the accuracy of our PH evaluations. Additionally, patients with extremely severe PH were excluded from this study due to their ineligibility for TAVR based on our selection criteria. This exclusion limits our insights into the outcomes of TAVR in this particularly high-risk patient subgroup, indicating a gap in current research that future studies could aim to address.

## 5. Conclusions

This study demonstrated that TAVR is both safe and effective in treating PNAR complicated by PH. We observed significant improvements in AR and reductions in PASP following TAVR, with no significant differences in the rate of postoperative adverse events between the two groups. Consequently, TAVR has emerged as a viable treatment option for this patient population. Looking ahead, the establishment of multi-center randomized controlled trials and long-term follow-up studies are needed to comprehensively evaluate the long-term efficacy and benefits of TAVR in patients with severe AR and PH. Such research efforts will be vital in solidifying the role of TAVR within this therapeutic domain.

## Data Availability

All data generated or analyzed during this study are included in this published article.

## References

[1] Jilaihawi H, Chen M, Webb J, Himbert D, Ruiz CE, Rodés-Cabau J (2016). A Bicuspid Aortic Valve Imaging Classification for the TAVR Era. *JACC. Cardiovascular Imaging*.

[2] Singh JP, Evans JC, Levy D, Larson MG, Freed LA, Fuller DL (1999). Prevalence and clinical determinants of mitral, tricuspid, and aortic regurgitation (the Framingham Heart Study). *The American Journal of Cardiology*.

[3] Markham R, Ghodsian M, Sharma R (2020). TAVR in Patients with Pure Aortic Regurgitation: Ready to Use?. *Current Cardiology Reports*.

[4] Dujardin KS, Enriquez-Sarano M, Schaff HV, Bailey KR, Seward JB, Tajik AJ (1999). Mortality and morbidity of aortic regurgitation in clinical practice. A long-term follow-up study. *Circulation*.

[5] Tarasoutchi F, Grinberg M, Spina GS, Sampaio RO, Cardoso LUF, Rossi EG (2003). Ten-year clinical laboratory follow-up after application of a symptom-based therapeutic strategy to patients with severe chronic aortic regurgitation of predominant rheumatic etiology. *Journal of the American College of Cardiology*.

[6] Lindman BR, Dweck MR, Lancellotti P, Généreux P, Piérard LA, O’Gara PT (2020). Management of Asymptomatic Severe Aortic Stenosis: Evolving Concepts in Timing of Valve Replacement. *JACC. Cardiovascular Imaging*.

[7] Gleason TG, Reardon MJ, Popma JJ, Deeb GM, Yakubov SJ, Lee JS (2018). 5-Year Outcomes of Self-Expanding Transcatheter Versus Surgical Aortic Valve Replacement in High-Risk Patients. *Journal of the American College of Cardiology*.

[8] Khandhar S, Varadarajan P, Turk R, Sampat U, Patel R, Kamath A (2009). Survival benefit of aortic valve replacement in patients with severe aortic regurgitation and pulmonary hypertension. *The Annals of Thoracic Surgery*.

[9] Rocha RV, Friedrich JO, Hong K, Lee J, Cheema A, Bagai A (2019). Aortic valve replacement with pulmonary hypertension: Meta-analysis of 70 676 patients. *Journal of Cardiac Surgery*.

[10] Maeder MT, Weber L, Rickli H (2022). Pulmonary hypertension in aortic valve stenosis. *Trends in Cardiovascular Medicine*.

[11] Miceli A, Varone E, Gilmanov D, Murzi M, Simeoni S, Concistrè G (2013). Impact of pulmonary hypertension on mortality after operation for isolated aortic valve stenosis. *International Journal of Cardiology*.

[12] Généreux P, Piazza N, Alu MC, Nazif T, Hahn RT, VARC-3 WRITING COMMITTEE (2021). Valve Academic Research Consortium 3: updated endpoint definitions for aortic valve clinical research. *European Heart Journal*.

[13] Akinseye OA, Pathak A, Ibebuogu UN (2018). Aortic Valve Regurgitation: A Comprehensive Review. *Current Problems in Cardiology*.

[14] Naidoo DP, Mitha AS, Vythilingum S, Chetty S (1991). Pulmonary hypertension in aortic regurgitation: early surgical outcome. *The Quarterly Journal of Medicine*.

[15] Arora S, Lahewala S, Zuzek Z, Thakkar S, Jani C, Jaswaney R (2021). Transcatheter aortic valve replacement in aortic regurgitation: The U.S. experience. *Catheterization and Cardiovascular Interventions: Official Journal of the Society for Cardiac Angiography & Interventions*.

